# Supplementary material for: Diagnostic Ability of Methods Depicting Distress of Tumor-Bearing Mice
Source: Animals (Basel). 2021 Jul 21;11(8):2155. doi: 10.3390/ani11082155 (PMC8388504; doi:10.3390/ani11082155)
Supplement: Supplementary file 1 [file animals-11-02155-s001.zip › animals-1277076-supplementary.pdf]

Table S1. Distress score on mice.

Observation	Score
**I Body weight**	
I-a decreased > 10% (compared to initial weight)	2
I-b decreased > 20% (compared to initial weight)	5
**II General condition**	
II-a tooth displacement, too long teeth	1 (A)
II-b fur dull, ruffled or untended	2
II-c eyes unclear or squinted	2
II-d untended orifices of the body	3
II-e abnormal posture	3
II-f dehydration	3
II-g short spasms or temporary paralysis symptoms	3
II-h persistent (>30′) cramping or paralysis	5
II-i abnormal respiratory sounds or animal feels cold	5
**III Spontaneous behavior**	
III-a the animal is passive or overactive	2
III -b pronounced apathy, hyperkinetic, or isolation	4
III -c squeaking due to pain	5
III -d self-mutilation	5
**IV Flight behavior after contact**	
IV-a animal is passive or overactive	2
IV-b distinct apathy or hyperkinetic	5
**V Process-specific criteria**	
V-a wound healing disorder	2
V-b opening of the sutures by biting	1 (B)
V-c local inflammation	2
V-d ascites	4
**Total score**	0–66

Score points are stated per line as soon as one criteria applies. Even with several positive results per line, there is no addition of the points per line.

Table S2. Consequences according to distress score.

Single Score	Total Score	Distress Level	Measures
A		mild	Anesthetize animal and shorten teeth. Document it.
B		mild	Inform the person in charge of the experiment. If necessary, anesthetize the animal and close the wound. Document it.
1		mild	Inform the person in charge of the experiment. A sufficient frequency of observation is necessary, consider treatment options and document it.
2–4		moderate	Inform the person in charge of the experiment. Daily observation of the animal is necessary, consider treatment options and document it.
5		severe	In agreement with the person in charge euthanasia (preferably painless after anesthesia) has to be performed. Document it.
	3–4	mild	Inform the person in charge of the experiment. Daily observation of the animal is necessary, consider treatment options and document it.
	5–15	moderate	Inform the person in charge of the experiment. Euthanasia or treating the animal plus daily observation of the animal is necessary. Document it.
	>15	severe	In agreement with the person in charge euthanasia (preferably painless after anesthesia) has to be performed. Document it.
